# Assessment of the Relationship Between Gastric-Acid Suppressants and the Risk of Esophageal Adenocarcinoma: A Systematic Review and Meta-Analysis

**DOI:** 10.1016/j.curtheres.2023.100692

**Published:** 2023-01-25

**Authors:** Karamali Kasiri, Catherine M.T. Sherwin, Sahar Rostamian, Saeid Heidari-Soureshjani

**Affiliations:** 1 Department of Pediatrics, Shahrekord University of Medical Sciences, Shahrekord, Iran; 2 Pediatric Clinical Pharmacology and Toxicology, Department of Pediatrics, Wright State University Boonshoft School of Medicine, Dayton Children's Hospital, Dayton, Ohio; 3 Shahrekord University of Medical Science, Student Research Committee, Shahrekord, Iran; 4 Modeling in Health Research Center, Shahrekord University of Medical Sciences, Shahrekord, Iran

**Keywords:** esophageal adenocarcinoma, gastric acid, histamine-2 receptor antagonists, oncology, proton pump inhibitors

## Abstract

**Background:**

Esophageal cancer is a cancerous tumor that develops in the esophagus. It is the 10th most common cancer and has a low survival rate. Esophageal adenocarcinoma (EAC) is increasing in incidence globally. Those with EAC are affected by Barrett's esophagus metaplasia, which is attributed to genetic predisposition and is more common in men. Studies suggest that gastric acid suppressants, like proton pump inhibitors and histamine-2 receptor antagonists, have anticancer properties and reduce EAC. However, other research has suggested that they are not cancer-protective, and the use of antisecretory drugs is a risk factor for developing EAC.

**Objective:**

This systematic review and meta-analysis investigated the properties and risk factors associated with using gastric acid suppressants in patients with EAC.

**Methods:**

This meta-analysis used the Preferred Reporting Items for Systematic Reviews and Meta-Analyses checklist. Information from selected articles, including the lead author's name, year of publication, study setting, sample size, and gender, was extracted and recorded into an Excel (Microsoft, Redmond, Washington) form. Statistical data included odds ratio, hazard ratio, and/or risk ratio, with a 95% CI associated with patients with EAC and receiving gastric acid suppressants. Data were compared with individuals not receiving treatment. Publication bias was assessed using Begg's and Egger's tests. Statistical analyzes used Stata 14.0 (Stata LLC, College Station, Texas).

**Results:**

The initial electronic literature search retrieved 3761 titles/abstracts. Extensive screening selected 20 articles for analysis. Odds ratios associated with EAC in the individuals using gastric acid suppressants were 0.77 (95% CI, 0.49–1.22; *P* = 0.274) and 0.67 (95% CI, 0.39–1.29; *P* = 0.240) for proton pump inhibitors and 1.02 (95% CI, 0.44-2.36; *P* = 0.967) for histamine-2 receptor antagonists.

**Conclusions:**

The results found that gastric acid suppressants do not have a protective role in EAC and are not risk factors. Future studies of confounding variables and risk factors are needed to understand what affects EAC development.

## Highlights

• Several studies have reported that treatment with gastric-acid suppressants can significantly decrease the risk of esophageal adenocarcinoma. However, other studies suggest antisecretory drugs are not cancer-protective and alternatively have concluded that the use of antisecretory drugs is a risk factor for developing esophageal adenocarcinoma (EAC).
• Studies have proposed several factors that may affect the relationship between gastric-acid suppressants, Barrett's esophagus, and EAC, but there is a general lack of consensus.
• There are inconclusive and varying results from historical studies related to the risk of EAC; however, this study concludes that gastric-acid suppressants do not have a protective effect and are not a risk factor for developing EAC.

## Introduction

Esophageal cancer is a malignant (cancerous) tumor that starts in the esophagus. It is the 10th most common cancer; even with surgery, chemotherapy, and radiation treatment, it has a low survival rate and can be fatal.^1^ Reportedly, it is more common in men than in women. Of concern is that the incidence of esophageal adenocarcinoma (EAC) has been rising globally. Histological categories are primarily squamous cell carcinoma and adenocarcinoma.^2^^,^^3^ Many of those with EAC are affected by Barrett's esophagus metaplasia, which results from prolonged tissue injury in the esophagus. Barrett's metaplasia is attributed to a genetic predisposition, gender, gastroesophageal reflux disease, smoking, alcohol consumption, high body mass index, and a poor diet lacking fruit and vegetables.^4^ In previous studies, antisecretory drugs, including proton pump inhibitors (PPIs) and histamine-2 receptor antagonists (H2RAs), were assumed to induce anticancer properties and reduce EAC development.

Some studies have reported that treatment with PPIs can significantly decrease the risk of EAC. In these patients, there is evidence that high-dose PPIs have a chemopreventive effect and can reduce the incidence of Barrett's esophagus metaplasia.^5^^,^^6^ In contrast, other studies have shown that the use of antisecretory drugs does not induce cancer-protective properties in these patients.^7^^,^^8^ Conversely, other studies have concluded that the use of antisecretory drugs is a risk factor that increases the occurrence of developing EAC.^9^, ^10^, ^11^, ^12^

Various risk factors have been identified and linked to the growing incidence of EAC. However, with the complexity of genetic risk factors and insufficient information available on other potential risk factors, it has not been easy to prevent and/or choose the appropriate treatment for those with EAC.^13^ Therefore, it is essential to investigate the disease further and reduce the knowledge gap concerning risk factors related to the disease. In addition, there is an overall need to find adequate health strategies that can be adopted to prevent the disease.

Previous meta-analyses have been conducted concerning this topic; however, to the best of our knowledge, none have comprehensively investigated antisecretory drugs as a preventive or potential risk factor associated with the incidence of EAC. As mentioned above, there are inconclusive and varying results from historical studies related to EAC. Therefore, in this systematic review and meta-analysis, we aimed to investigate the properties and the risk factors associated with using PPI and H2RA therapies for patients with EAC.

## Methods

### Data sources and search strategy

This meta-analysis used the Preferred Reporting Items for Systematic Reviews and Meta-Analyses checklist as a guideline (http://prisma-statement.org/prismastatement/Checklist.aspx). A systematic literature search was undertaken on June 28, 2022, using PubMed, Web of Science, Embase, and Scopus databases. The following keywords were used for searching in the databases: ((*esophageal adenocarcinoma* OR *esophageal cancer* OR *esophageal squamous-cell carcinoma*) AND (*acid suppress* OR *proton pump inhibitor* OR *PPIs* OR *histamine receptor antagonists* OR *H2RAs* OR *H2 blocker* OR *omeprazole* OR *esomeprazole* OR *dexlansoprazole* OR *lansoprazole* OR *pantoprazole* OR *cimetidine* OR *ranitidine* OR *rabeprazole* OR *azacitidine* OR *famotidine* OR *lafutidine*)).

### Study selection

**Figure 1:**
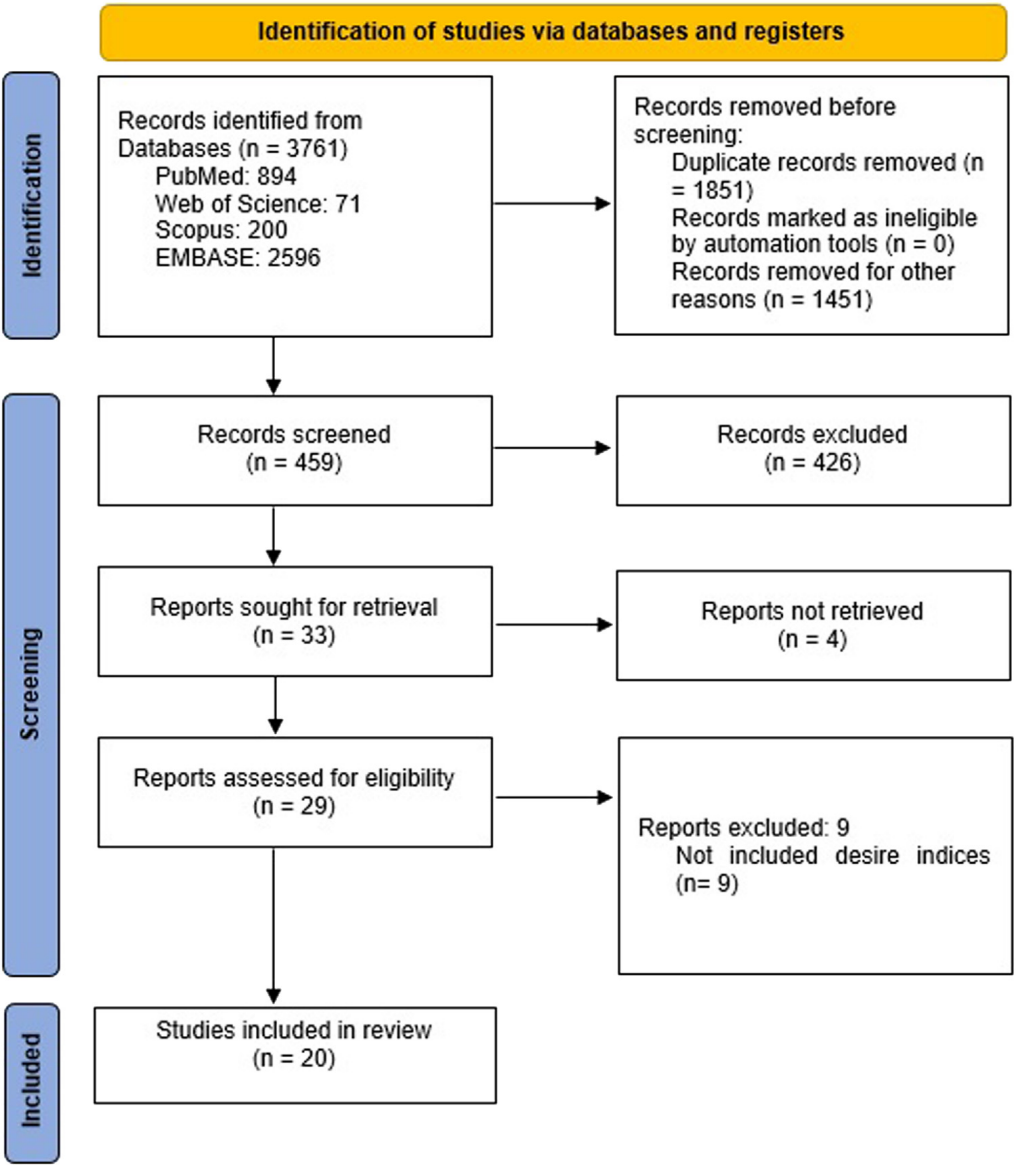
The flowchart shows the studies included in the meta-analysis.

After entering the key words mentioned into the databases, relevant records were imported into EndNote X8 (Clarivate Analytics, Philadelphia, Pennsylvania). This software was used to organize the articles and identify and remove duplicate records. Two researchers independently identified the studies by reviewing the titles and abstracts of the publications based on the selected inclusion and exclusion criteria. The inclusion criteria included the study's goals and looked for publications that addressed the association between gastric acid suppressants and the risk of developing EAC. The exclusion criteria included removing non-English language studies and any studies where a complete publication was unavailable. After gathering all eligible full-text articles to be included in the study, they were independently reviewed. If there was any conflict during the review of the studies, a discussion occurred to reach a consensus. Finally, a third team member was asked to help decide if no consensus could be reached. Figure 1 outlines the strategy flowchart used to identify and screen the literature.

### Data extraction and quality assessment

Two researchers independently screened the records and extracted specific data to be included in this review. Information was collected from the selected articles, including the lead author's name, year of publication, study setting, sample size, patient gender, the mean age of the study population, length of follow-up, duration of treatment (receiving antisecretory drugs), and adjusted variables. In addition, all data were extracted and recorded into an Excel (Microsoft, Redmond, Washington) form. Statistical information, including odds ratio (OR), hazard ratio, and/or risk ratio, with a 95% CI associated with patients who had EAC and were receiving gastric acid suppressants, were compared with individuals who did not receive this type of treatment.

### Evaluating the quality of the studies

Quality assessment and risk of bias assessment tools pertinent to observational studies were determined using the Newcastle-Ottawa scale.^14^ Several items, such as selection, comparability, and exposure/outcome, determined these results. Studies with at least a score of 7 on this scale were high-quality studies. The quality and the risk of biased estimates of treatment used in the randomized controlled trial articles were measured by the Jadad scale.^15^^,^^16^ Jadad scores were from 0 to 5, and those clinical trials scoring 3 points or above were considered indications of decent quality studies.

### Statistical analysis

This systematic review and meta-analysis used ORs to measure the relationship between those receiving gastric acid suppressants and the risk of developing EAC. The effect size of the association between study exposure and the relevant outcome was conducted using OR with 95% CI. The random-effect models from the meta-analysis were used to estimate the overall summary. Forest plots were used to illustrate the graphical representations of the individual OR and summary estimates. According to an a priori decision, subgroup analyses used factors such as geographic regions, which included Europe, North America, Asia, and Australia. The sample size of studies was (˃1000 vs ≤1000); duration of treatment (receiving antisecretory drugs) was (˃5 years vs ≤5 years); study designs were case-control, cohort, or randomized controlled trials; and the study period was between 2000-2010 and 2011-2022. The quality of the studies was determined to be medium, good, or excellent.

Heterogeneity in the studies was determined using the Cochran χ^2^ test (reported by χ^2^ with a *P* < 0.1 level of significance) and also included the *I*^2^ statistic. A series of sensitivity analyses were conducted to evaluate the findings' robustness and to characterize potential statistical heterogeneity sources. Initially, the effect of individual studies on the summary estimates was determined using sensitivity analyses. Subsequently, pooled estimates were re-calculated after deleting 1 study after each run. The meta-regression analysis determined the differences between studies and the observed effect size. Any potential publication bias was detected using Begg's and Egger's tests. All statistical analyzes were conducted using Stata 14.0 (Stata LLC, College Station, Texas), where *P* < 0.05 was considered statistically significant.

## Results

### Search results and study characteristics of selected studies

Figure 1 illustrates the Preferred Reporting Items for Systematic Reviews and Meta-Analyses flow diagram and outlines the search strategy. The initial electronic literature search retrieved 3761 titles and abstracts. Duplicate publications were omitted (n = 1851), and 1451 were removed based on the study inclusion and exclusion criteria. In the screening process, there were 459 records. A thorough review of the remaining records identified irrelevant articles that did not fit the study's focus and therefore were excluded. This left 33 records to be retrieved for more advanced screening. Additional records were excluded (n = 13). Three records were removed because of an inability to retrieve the full text of articles.^17^, ^18^, ^19^ Nine records did not include data consistent with this research's goal.^12^^,^^20^, ^21^, ^22^, ^23^, ^24^, ^25^, ^26^, ^27^ One study did not contain results that were useful.^28^ Finally, 20 articles were selected for the final evaluation to investigate the association between gastric acid suppressants and the risk of developing EAC.^9^, ^10^, ^11^^,^^29^, ^30^, ^31^, ^32^, ^33^, ^34^, ^35^, ^36^, ^37^, ^38^, ^39^, ^40^, ^41^, ^42^, ^43^, ^44^, ^45^

### Characteristics of selected studies regarding the association between gastric acid suppressants and the risk of developing EAC

From the 20 studies in this analysis, there were 1,155,699 patients. Of these, 11 studies used a cohort design, which included 1,125,437 patients^10^^,^^11^^,^^29^, ^30^, ^31^^,^^34^^,^^35^^,^^38^^,^^39^^,^^42^^,^^45^ and 8 studies had a case–control design, with 27,727 participants.^9^^,^^32^^,^^33^^,^^36^^,^^40^^,^^41^^,^^43^^,^^44^ One study was conducted as an randomized controlled trial and included 2,535 patients.^37^ The articles selected for this systematic review and meta-analysis were published between 2004 and 2022. The studies were geographically diverse, comprising 9 conducted in North America, (n = 38,469 participants).^10^^,^^32^^,^^34^^,^^39^^,^^40^^,^^42^, ^43^, ^44^, ^45^ Two studies were from Asia (eg, Japan and South Korea) and involved 312,848 participants.^11^^,^^29^ Eight studies included 804,032 participants from Europe (eg, United Kingdom, Netherlands, Denmark, and Sweden).^9^^,^^30^^,^^31^^,^^33^^,^^36^, ^37^, ^38^^,^^41^ Finally, 1 was undertaken in Australia, comprising 350 patients.^35^ The mean (SD) follow-up of the participants was 81.38 (32.71) months (Tables 1 and 2).

**Table 1:** Characteristics of included studies for reviewing the relationship between gastric acid suppressants and esophageal adenocarcinoma

Publication lead author	Year	Study setting	Study design	Male/Female	Patientage, y	Sample size	Odds ratio for PPIs	Odds ratio for H2RAs	Quality of the studies
Tan^44^	2018	United States	Case-control	100/0	64.8	1098	0.59 (0.35–0.99)	0.7 (0.5–0.99)	7
Krishnamoorthi^39^	2016	United States	Cohort	64/36	63	9660	0.69 (0.52–0.92)	–	9
Nguyen^42^	2009	United States	Cohort	94/6	61	344	0.39 (0.19–0.80)	–	8
Kastelein^38^	2013	Netherlands	Cohort	71/29	61	540	0.21 (0.07–0.66)	0.83 (0.11–6.03)	8
Choi^11^	2022	Korea	Cohort	92.98/7.02	63	4055	13.23 (10.25–17.06)	4.34 (3.67–5.14)	8
Cooper^31^	2006	United Kingdom	Cohort	59.57/40.43	61	188	0.33 (0.07–0.97)	–	5
Garcia Rodriguez^9^	2006	United Kingdom	Case-control	79/21		1809	0.84 (0.48–1.5)	1.2 (0.78–1.84)	8
Gaddam^34^	2014	United States	Cohort	87.8/12.2	60.9	3635	0.41 (0.22–0.75)	0.58 (0.39–88)	3
Nguyen^43^	2010	United States	Case-control	97/3	65	812	1.50 (0.61–3.66)	–	7
Masclee^41^	2015	United Kingdom	Case-control	63/37	64.8	696	0.90 (0.30–2.30)	–	8
Loomans-Kropp^40^	2021	United States	Case-control	NA	68	21373	0.30 (0.20-0.44)	–	6
de Jonge^33^	2006	Netherlands	Case-control	74/26	62	335	0.09 (0.05–0.20)	–	7
Thota^45^	2017	United States	Cohort	72/28	59	1025	0.49 (0.27–0.89)	0.62 (0.27–1.41)	9
Jung^10^	2011	United States	Cohort	69/31	63	355	0.23 (0.03–1.72)	–	9
Brusselaers^30^	2018	Sweden	Cohort	41.5/58.5	55	796492	3.93 (3.63–4.24)	0.39 (0.04–1.40)	6
Hillman^35^	2004	Australia	Cohort	71/29	58	350	0.05 (0.006–0.36)	–	8
Hvid-Jensen^36^	2014	Denmark	Case-control	66.5/33.5	62.6	1437	1.90 (0.70–4.90)	–	6
Jankowski^37^	2018	United Kingdom	RCT	79.5/20.5	57	2535	1.04 (0.67–1.61)	–	3*
Arai^29^	2022	Japan	Cohort	81.58/18.42	70	308793	0.66 (0.42–1.04)	–	8
Crane^32^	2007	United States	Case-control	83/17	70	167	4 (0.4–36)	1.8 (0.5–6.0)	5

NA = not available; PPI = proton pump inhibitor; RCT = randomized controlled trial.

⁎ The Jadad scale assessed this study, and the other was assessed by the Newcastle-Ottawa scale.

**Table 2:** Adjusted variables in assessment relationship between gastric acid suppressants and esophageal adenocarcinoma.

Publication lead author	Year	Adjustment
Tan^44^	2018	Age at BE diagnosis, BMI, smoking status, H2RA use, number of EGDs after BE diagnosis, aspirin use, statin use, and NSAID consumption
Krishnamoorthi^39^	2016	Age, sex, BMI, hiatal hernia, smoking, diabetes mellitus-type 2, using PPI, NSAIDs, metformin, insulin, other anti-diabetes medications, and statin
Nguyen^42^	2009	Age at BE diagnosis, sex, BE length
Kastelein^38^	2013	Age, sex, histology, BE length, baseline PPI use, use of other medications
Choi^11^	2022	Sex, age, income, and residence
Cooper^31^	2006	NA
Garcia Rodriguez^9^	2006	Age, sex, calendar year, alcohol use, smoking, BMI, gastroesophageal reflux, hiatal hernia, peptic ulcer, and dyspepsia
Gaddam^34^	2014	Male gender, age, smoking, Caucasian race, BE length, H2RA use, aspirin, and NSAID use
Nguyen^43^	2010	Race, number of outpatient encounters, noncancer disease comorbidity index, filled prescriptions for NSAIDs/statins, and aspirin
Masclee^41^	2015	Duration of follow-up since BE diagnosis
Loomans-Kropp^40^	2021	Age, inflammatory bowel disease, history of gastroesophageal reflux disease, and diabetes with complications
de Jonge^33^	2006	Age, sex, education level, alcohol use, smoking, and reflux
Thota^45^	2017	BE length, age, sex, and hiatal hernia size
Jung^10^	2011	Age, sex
Brusselaers^30^	2018	NA
Hillman^35^	2004	Age, sex, use of aspirin or NSAIDs, and presence of macroscopic markers
Hvid-Jensen^36^	2014	Presence of BE with low-grade dysplasia, gender, use of statins, NSAIDs, low-dose aspirin, and high-dose aspirin, and antidiabetes drugs
Jankowski^37^	2018	Age, BE length, intestinal metaplasia
Arai^29^	2022	Age, sex, smoking with/without gastroesophageal reflux disease, and Charlson comorbidity index scores
Crane^32^	2007	NA

BE = Barrett's esophagus; BMI = body mass index; EGD = esophagogastroduodenoscopy; H2RA = histamine 2 receptor antagonist; NA = not applicable; NSAID = nonsteroidal anti-inflammatory drug; PPI = proton pump inhibitor.

### Relationship between gastric acid suppressants and EAC

**Figure 2:**
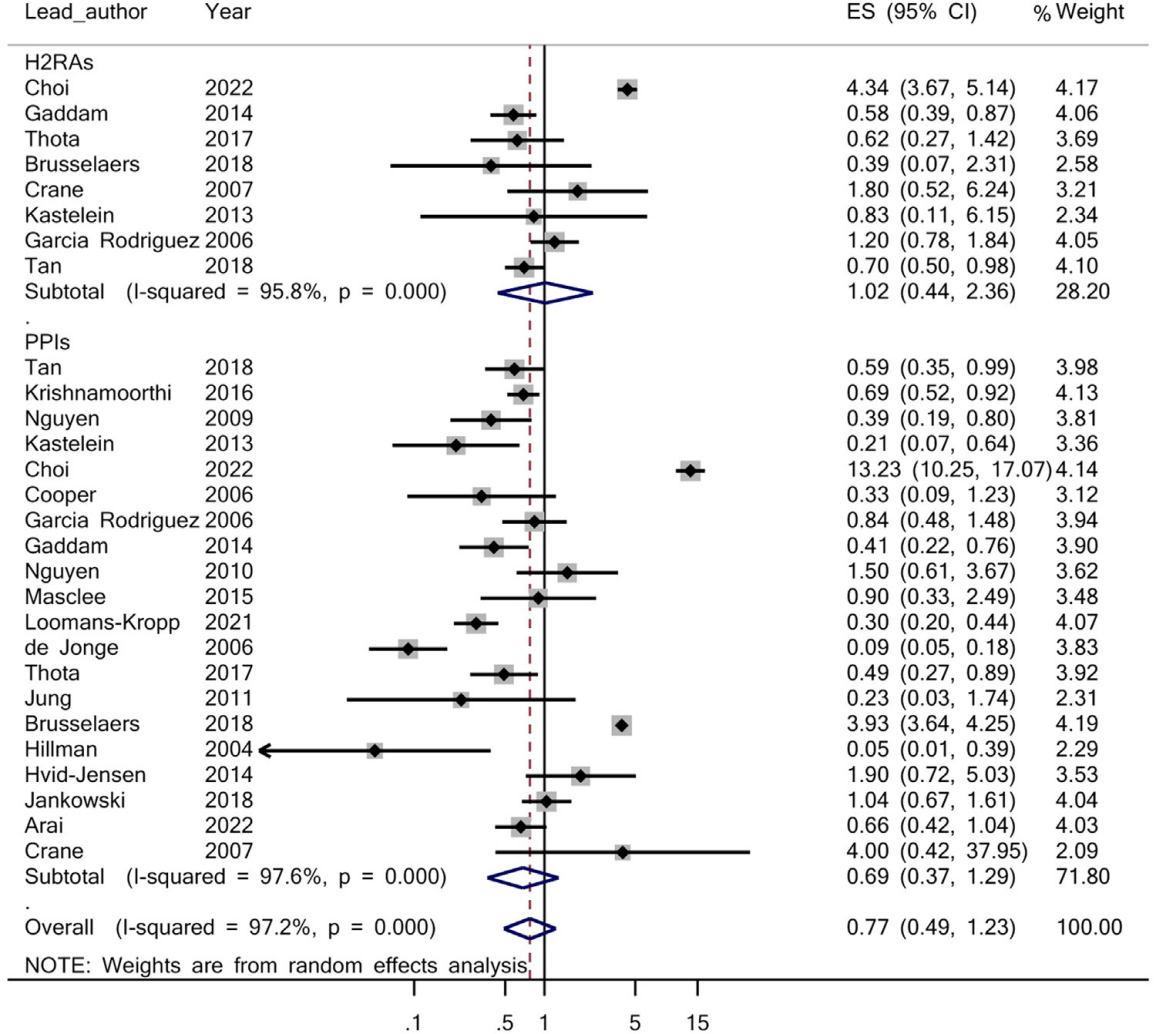
Forest plot of the relationship between gastric acid suppressants and esophageal adenocarcinoma. ES = Effect size; HR2A = histamine-2 receptor antagonist; PPI = proton pump inhibitor.

Based on the adjusted OR (Table 3) obtained from each study in the meta-analysis, when compared with the group not receiving antisecretory drugs, the OR of EAC in the recipients of antisecretory drugs group was 0.77 (95% CI, 0.49–1.22; *P* = 0.274). Similarly, the OR of EAC in the individuals from the PPIs drug group was 0.67 (95% CI, 0.39–1.29; *P* = 0.240) and in those in the H2RAs drug group this was 1.02 (95% CI, 0.44–2.36; *P* = 0.967) (Figure 2).

**Table 3:** Sensitivity analysis for the assessment of the relationship between gastric acid suppressants and esophageal adenocarcinoma

First author	Year	Drug type	Estimated odds ratio (95% CI)
Hillman^35^	2004	PPI	0.83 (0.52–1.310
Cooper^31^	2006	PPI	0.80 (0.50–1.270
Nguyen^42^	2009	PPI	0.80 (0.50–1.27)
Nguyen^43^	2010	PPI	0.75 (0.47–1.21)
Kastelein^38^	2013	PPI	0.81(0.51–1.29)
Gaddam^34^	2014	PPI	0.79(0.50–1.27)
Hvid-Jensen^36^	2014	PPI	0.75(0.47–1.20)
Masclee^41^	2015	PPI	0.77(0.48–1.23)
Krishnamoorthi^39^	2016	PPI	0.78(0.49–1.24)
Thota^45^	2017	PPI	0.79(0.49–1.26)
Jankowski^37^	2018	PPI	0.76(0.48–1.23)
Tan^44^	2018	PPI	0.78(0.49–1.25)
Loomans-Kropp^40^	2021	PPI	0.81(0.52–1.27)
Arai^29^	2022	PPI	0.78(0.49–1.24)
de Jonge^33^	2006	PPI	0.85(0.54–1.33)
Garcia Rodriguez^9^	2006	PPI	0.77(0.48–1.23)
Crane^32^	2007	PPI	0.75(0.47–1.19)
Jung^10^	2011	PPI	0.80 (0.50–1.27)
Brusselaers^30^	2018	PPI	0.71(0.42–1.21)
Choi^11^	2022	PPI	0.69(0.44–1.08)
Kastelein^38^	2013	H2RA	0.77(0.49–1.23)
Gaddam^34^	2014	H2RA	0.78(0.49–1.25)
Thota^45^	2017	H2RA	0.78(0.49–1.25)
Tan^44^	2018	H2RA	0.76 (0.47–1.22)
Garcia Rodriguez^9^	2006	H2RA	0.76 (0.47–1.22)
Crane^32^	2007	H2RA	0.75(0.47–1.20)
Brusselaers^30^	2018	H2RA	0.79 (0.49–1.25)
Choi^11^	2022	H2RA	0.71 (0.42–1.20)
Combined			0.77 (0.49–1.22)

H2RA = histamine-2 receptor antagonist; PPI = proton-pump inhibitor.

### Meta-regression model and sensitivity analysis

**Figure 3:**
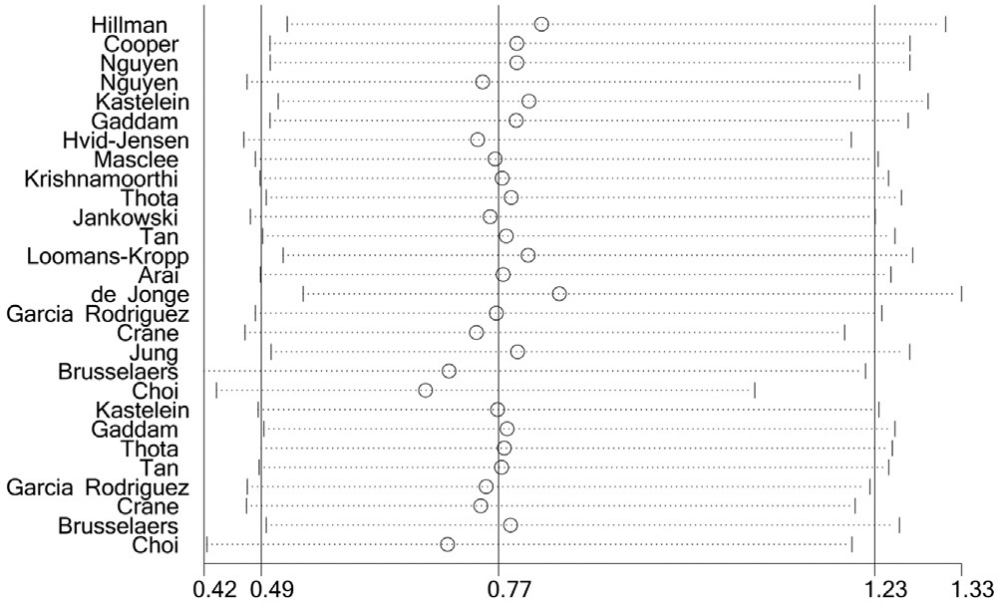
Sensitivity analysis for the assessment of the relationship between gastric acid suppressants and esophageal adenocarcinoma.

There was significant heterogeneity identified within the results from the meta-analysis (χ^2^ = 972.92; df = 27; *P* ≤ 0.001; *I*^2^ = 97.2%). To further investigate the source of heterogeneity, a meta-regression model was performed, which considered variables such as year, follow-up time, study design, sample size, study period, the quality of the study, duration of treatment and geographic region. The meta-regression analysis results show no significant source of heterogeneity (*P* > 0.10). During each run, a sensitivity analysis was done by excluding each study from the analysis 1 by 1. However, the estimated OR did not change significantly, indicating the robustness of the meta-analysis results (Table 3 and Figure 3).

### Subgroup analysis

A subgroup analysis was performed to determine the association between gastric acid suppressants and EAC by assessing the study design, sample size, study period, study quality, and geographic location. The OR of EAC in those that receiving gastric acid suppressants was 0.74 (95% CI, 0.45–1.22; *P* = 0.245) from the case–control studies, 0.76 (95% CI, 0.0.43–1.35; *P* = 0.353) in the cohort studies, and 1.04 (95% CI, 0.67–1.61; *P* = 0.861) from the randomized controlled trials. The results of the subgroup analysis evaluating study design, sample size, quality of studies, study period, geographic location, and duration of treatment (receiving antisecretory drugs) are detailed in Table 4.

**Table 4:** Subgroup analysis of the association between gastric acid suppressants and esophageal adenocarcinoma.

Characteristic		Study No.	Odds ratio (95% CI)	*P* value
Study type	Case-control	11	0.74 (0.45–1.22)	0.245
	Cohort	16	0.76 (0.43–1.35)	0.353
	Randomized controlled trial	1	1.04 (0.67–1.61)	0.861
Geographic location	North America	13	0.58 (0.45–0.74)	0.001
	Europe	11	0.71 (0.31–1.60)	0.407
	Asia	3	0.77 (0.49–1.22)	0.057
	Australia	1	0.05 (0.006–0.038)	0.004
Study period	2000–2010	9	0.57 (0.26–1.23)	0.152
	2011–2022	19	0.88 (0.52–1.50)	0.652
Quality of the studies	Medium	6	0.72 (0.44–1.180	0.198
	Good	8	0.69 (0.22–2.07)	0.514
	Excellent	14	0.78 (0.37–1.64)	0.516
Sample size	>1000	17	1.01 (0.59–1.72)	0.961
	≤1000	11	0.45 (0.21–0.96)	0.040
Duration of treatment (receiving antisecretory drugs)	>5 years	11	0.81 (0.49–1.29)	0.411
	≤5 years	17	0.63 (0.25–1.43)	0.155

### Evaluation of publication bias related to gastric acid suppressants and EAC

**Figure 4:**
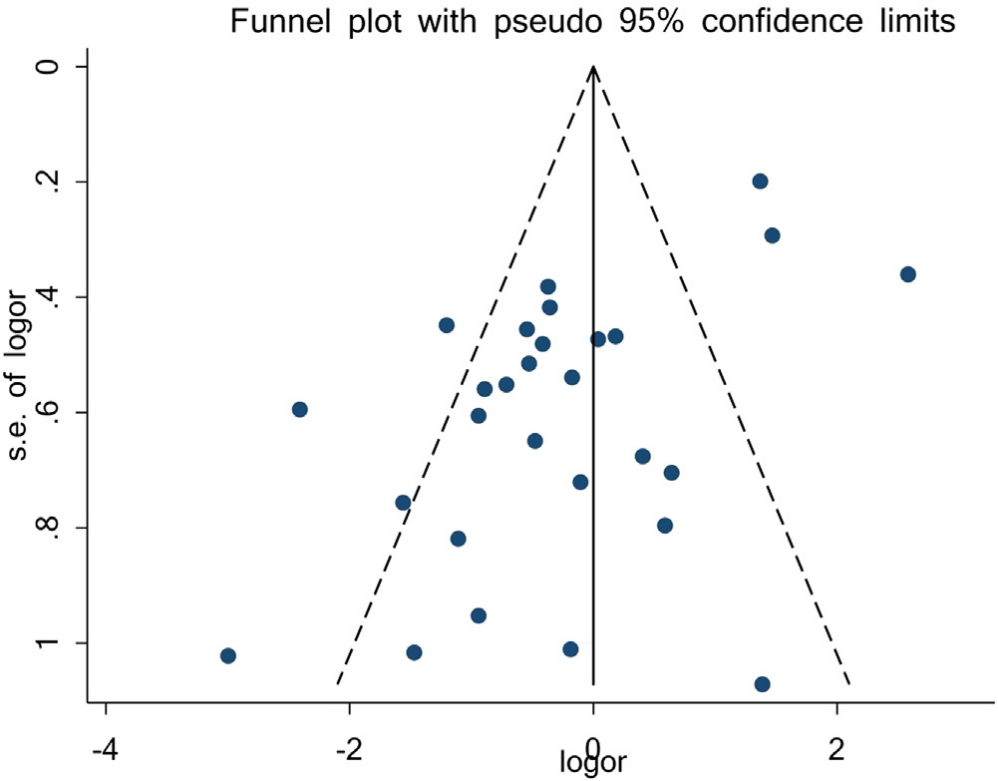
Funnel diagram to evaluate publication bias in the relation between gastric acid suppressants and esophageal adenocarcinoma.

Following the previous statistical assessment of the articles' data, there was evidence of publication bias. This was suspected upon evaluating the reported relationship between gastric acid suppressants and EAC. Statistical tests were conducted to evaluate potential publication bias in the reported studies. Application of Egger's test (*P* = 0.001) produced statistically significant results. However, the results obtained were insignificant when using Begg's test (*P* = 0.061) (Figure 4). Therefore, published studies that report on the relationship between gastric acid suppressants and EAC are significantly associated with publication bias, which can influence the meta-analysis results.^46^^,^^47^ The metatrim command (which performs the Duval and Tweedie nonparametric "trim and fill" method to assess publication bias in a meta-analysis) was used to consider the effect size of the missing studies and to estimate the final OR of 0.77 (95% CI, 0.49–1.22), which did not change.

## Discussion

This systematic review and meta-analysis investigated the relationship between gastric acid suppressants and the risk of developing EAC. Results showed that when comparing to groups that did not receive gastric acid suppressants, the OR for EAC in the group taking gastric acid suppressants was 0.77 (95% CI, 0.49–1.22; *P* = 0.274). In addition, the OR of EAC in the PPI group was also 0.67 (95% CI, 0.39–1.29; *P* = 0.240). These results are consistent with those outlined by Hu et al,^7^ who reported that PPI use is not associated with an increased risk of EAC in patients with Barrett's esophagus metaplasia (OR = 0.43; 95% CI, 0.17–1.08).

To date, no comprehensive published meta-analysis studies have investigated the effect of H2RA use on EAC. In this study, the OR of EAC in patients in the H2RAs group was 1.02 (95% CI, 0.44–2.36; *P* = 0.967). Unfortunately, there was insufficient evidence to draw a reasonable conclusion from this study. However, other reports have reported an association between H2RA therapy and the risk of EAC, although these studies did not indicate any statistically significant effects.^6^

Contrary to the results of this study, Chen et al,^4^ in their meta-analysis, reported that PPI therapy was related to a decrease in the risk of Barrett's esophagus progression, which was the most substantial risk for to EAC (OR = 0.47; 95% CI, 0.32–0.71). EAC might develop through the advancement of metaplasia to dysplasia to invasive carcinoma. An additional study evaluated the relationship between gastric acid suppressants and the risk of EAC in patients with Barrett's esophagus. They stated that PPI consumption was associated with a 71% reduction in the risk of EAC in individuals with Barrett's esophagus (OR = 0.29; 95% CI, 0.12–0.79).^6^ Different studies have proposed several factors that may affect the relationship between gastric acid suppressants, Barrett's esophagus, and EAC, contributing to the general lack of consensus.

Overall, there is a lack of control over potential confounding factors within these studies. This is among the significant concerns related to investigations into this disease. Several etiological underlying factors have been proposed to have a role in the occurrence of this disease. For example, age, sex, a diet low in fruits and vegetables, alcohol, smoking, genetic factors, and medication use.^48^, ^49^, ^50^ Aside from those factors, variability in results may be related to the study methods, how patients are included, and subsequent available data and calculated statistics. For example, data deficiencies may be caused by a small sample size, poor allocation to therapy groups, and a short follow-up period.

Several potential limitations exist in the current systematic review and meta-analysis that should be considered when interpreting the results. For example, within the literature screening, only 1 randomized controlled trial was included in the analysis, and the rest of the data came from observational studies. This analysis planned to investigate various confounding factors that have been reported to be associated with gastric acid suppressants and EAC. Factors that were to be included in the analysis included smoking, alcohol consumption, and body mass index. It has also been suggested that Barrett's esophagus length (short, long, or very long segments) can be a risk factor for developing dysplasia and adenocarcinoma. Data for some risk factors, such as Barrett's esophagus length, was limited in the articles reviewed. In addition, data were missing in other studies, and there was no consistency in the reported confounding factors. This means that some results may be biased in their reported effects between the potential risk and disease relationship (ie, risk factors or exposure are distorted because they are mixed with other variables). Finally, drug–drug and drug–disease interactions could cause adverse effects with the antacid compounds, PPIs, and EAC. However, there have only been limited investigations that have considered including these as confounding factors.

## Conclusions

The results determined no statistically significant association between gastric acid suppressants and the risk of developing EAC. Based on the data analysis for PPIs, they do not play a protective role for patients with EAC. Additionally, they were not a risk factor for this disease. However, more randomized controlled trials are needed to investigate potential confounding factors and better assess the relationship between Barrett's esophagus length as a risk factor for developing dysplasia and adenocarcinoma. Finally, there were only limited data that investigated the effect of H2RA use and the risk of EAC. Due to this, we could not draw robust conclusions related to H2RAs and EAC. The incidence of EAC has been growing globally, and the development of this malignant tumor in the esophagus is the 10th most common cause of cancer. It has a low survival rate and can be fatal. With the paucity of research in this area, we urgently need future studies with consistent and robust data that report confounding factors that offer clinically valuable conclusions leading to improved treatment and reduced risk of developing EAC.

## Conflicts of Interest

The authors have indicated that there is no conflict of interest regarding the content of this article.
